# Supplementary material for: Toll/interleukin-1 receptor (TIR) domain-containing proteins have NAD-RNA decapping activity
Source: Nat Commun. 2024 Mar 13;15:2261. doi: 10.1038/s41467-024-46499-y (PMC10937652; doi:10.1038/s41467-024-46499-y)
Supplement: Supplementary file 1 — Supplementary Information [file 41467_2024_46499_MOESM1_ESM.docx]

**Toll/interleukin-1 receptor (TIR) domain-containing proteins have NAD-RNA decapping activity**

Xufeng Wang^1, 2, 3, †^, Dongli Yu^4, 5, 6, †^, Jiancheng Yu^7^, Hao Hu^1, 2, 3^, Runlai Hang^1, 2, 3^, Zachary Amador^3^, Qi Chen^7, 8^, Jijie Chai^4, 5^, and Xuemei Chen^1, 2 *^

^1^ State Key Laboratory for Protein and Plant Gene Research, Peking-Tsinghua Joint Center for Life Sciences, School of Life Sciences, Peking University, Beijing, 100871, China

^2^ Beijing Advanced Center of RNA Biology (BEACON), Peking University, Beijing, 100871, China

^3^ Department of Botany and Plant Sciences, Institute of Integrative Genome Biology, University of California, Riverside, CA 92521, USA

^4^ Institute of Biochemistry, University of Cologne, Cologne, 50674, Germany

^5^ Max Planck Institute for Plant Breeding Research, Cologne, 50829, Germany

^6^ Dana-Farber Cancer Institute, Harvard Medical School, Boston, MA 02215, USA

^7^ Department of human genetics, University of Utah School of Medicine, Salt Lake City, UT 84112, USA

^8^ Molecular Medicine Program, Division of Urology, Department of Surgery, University of Utah School of Medicine, Salt Lake City, UT 84112, USA

^*^ Address correspondence to xuemei.chen@pku.edu.cn

^†^ These authors contributed equally to this work.


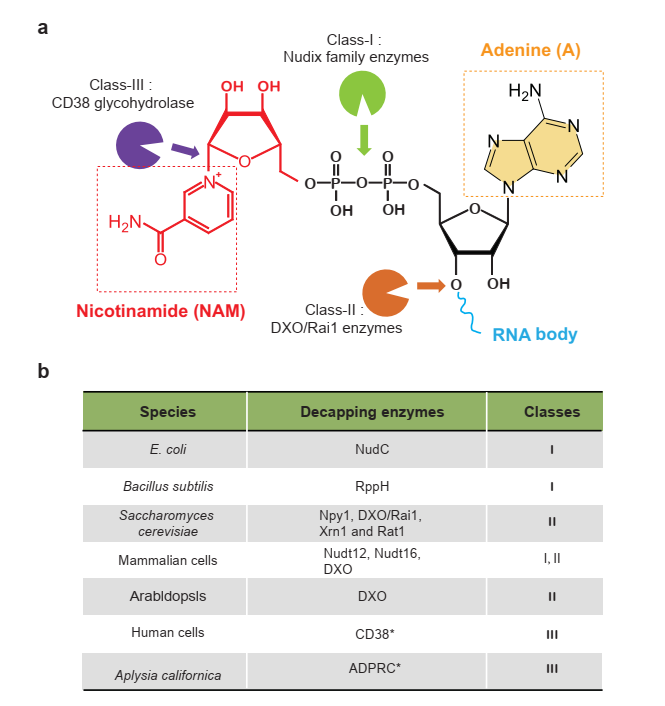


**Supplementary Fig. 1: a** Structure of NAD-capped RNA (NAD-RNA) and the decapping enzymes that can cleave or remove the NAD cap. The Nudix (nucleoside diphosphate linked to a variable moiety X) enzymes cleave the pyrophosphate bond to release nicotinamide mononucleotide (NMN). The DXO/Rai1 family enzymes cleave the phosphodiester linkage to release NAD^+^. The human CD38 glycohydrolase cleaves the *β-*N-glycosidic bond to release the nicotinamide (NAM) moiety. **b** Summary of NAD-RNA decapping enzymes from various organisms. * Only *in vitro* decapping activities were reported for CD38 and ADPRC.


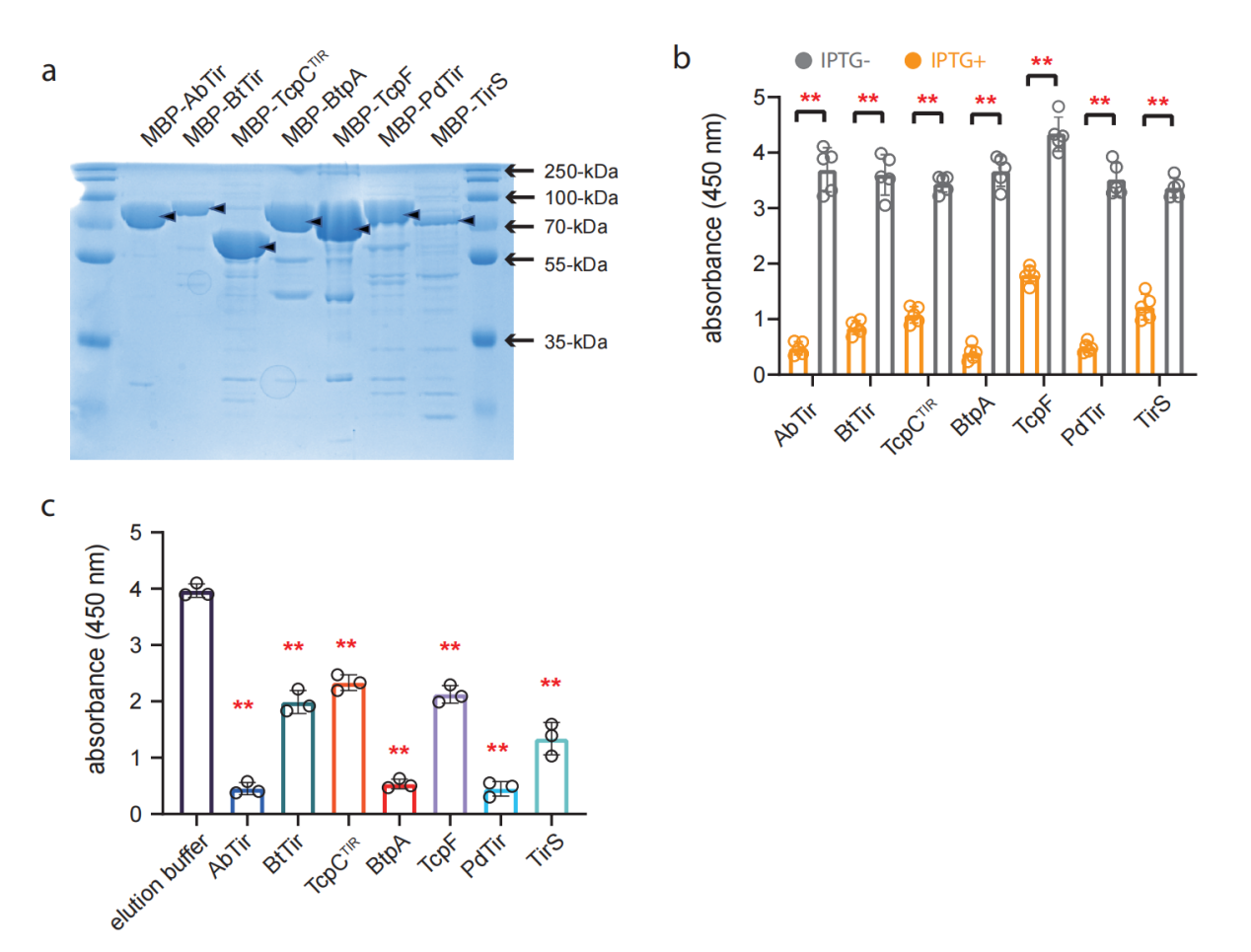


**Supplementary Fig. 2:** The NADase activities of bacterial TIR domain-containing proteins. **a** An SDS-PAGE gel showing the purified recombinant TIR domain-containing proteins. All proteins were expressed in, and purified from, BL21 *E. coli* cells. The proteins were separated in a 15% SDS-PAGE gel and stained with Coomassie Brilliant Blue. Arrowheads indicate the recombinant proteins. **b** Endogenous NAD^+^ contents in *E. coli* cells expressing the TIR domain-containing proteins (IPTG+) or not (IPTG-). Error bars represent mean ± SD, which was calculated from five biological replicates (n = 5). **c** *In vitro* NADase assays in which purified TIR domain-containing proteins were incubated with free NAD^+^. Error bars represent mean ± SD, which was calculated from three independent experiments (n = 3). Measurement of NAD^+^ levels in (b and c) were performed using the NAD/NADH Quantitation Kit. The Y-axis stands for the absorption values at 450 nm, which reflect NAD^+^ levels. [**] *P* ≤ 0.01 (calculated by the non-parametric Mann-Whitney U-test, two-sided). Exact *P*-values and source data are provided as a Source Data file.


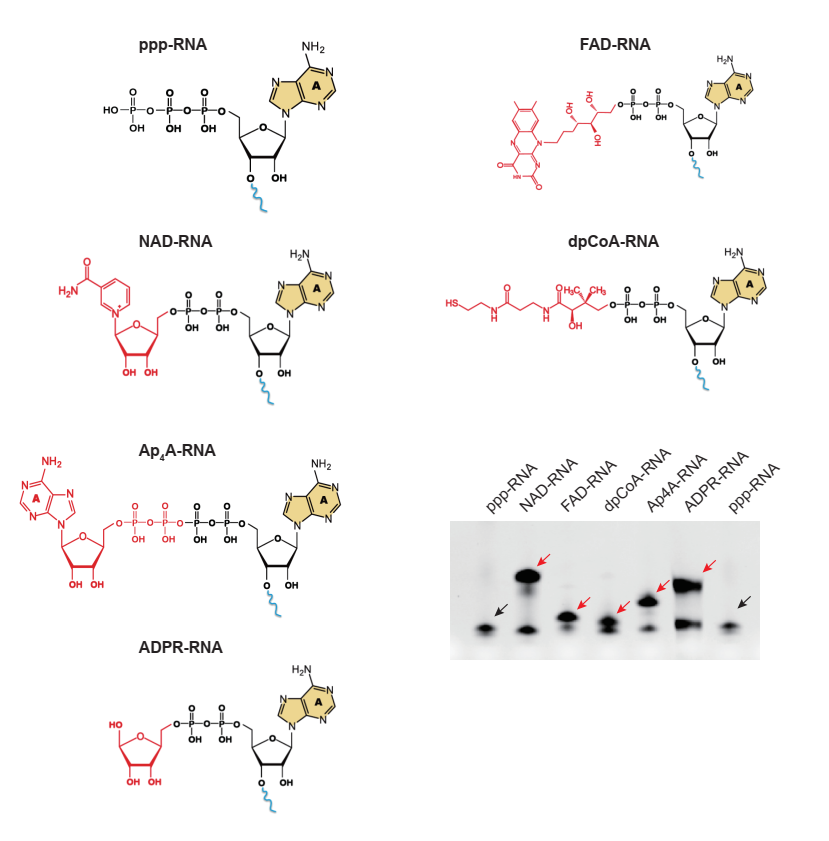


**Supplementary Fig. 3:** Diagrams of various cap structures and an acryloylaminophenyl boronic acid (APB) gel showing the migration of 32-nucleotide (nt), *in vitro* transcribed RNAs with various non-canonical RNA-caps at the 5’-end. Red arrows indicate RNAs with non-canonical caps. Source data are provided as a Source Data file.


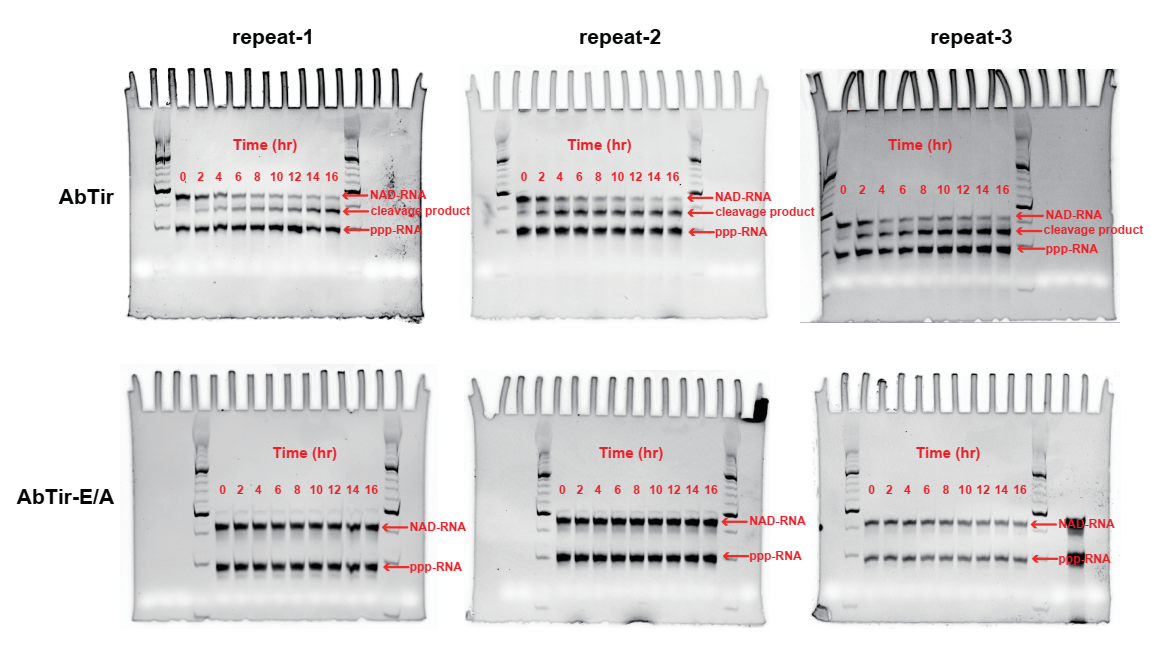


**Supplementary Fig. 4:** Time-courses of NAD-RNA deNAMing assays with wild-type AbTir and its catalytic mutant AbTir-E/A. An *in vitro* transcribed NAD-RNA was incubated with AbTir (top panel) or its catalytic mutant AbTir-E/A (bottom panel), and the products at the indicated time points were resolved on denaturing APB gels. Three independent experiments (repeats) were performed for the analysis of reaction kinetics in **Fig. 2b**.


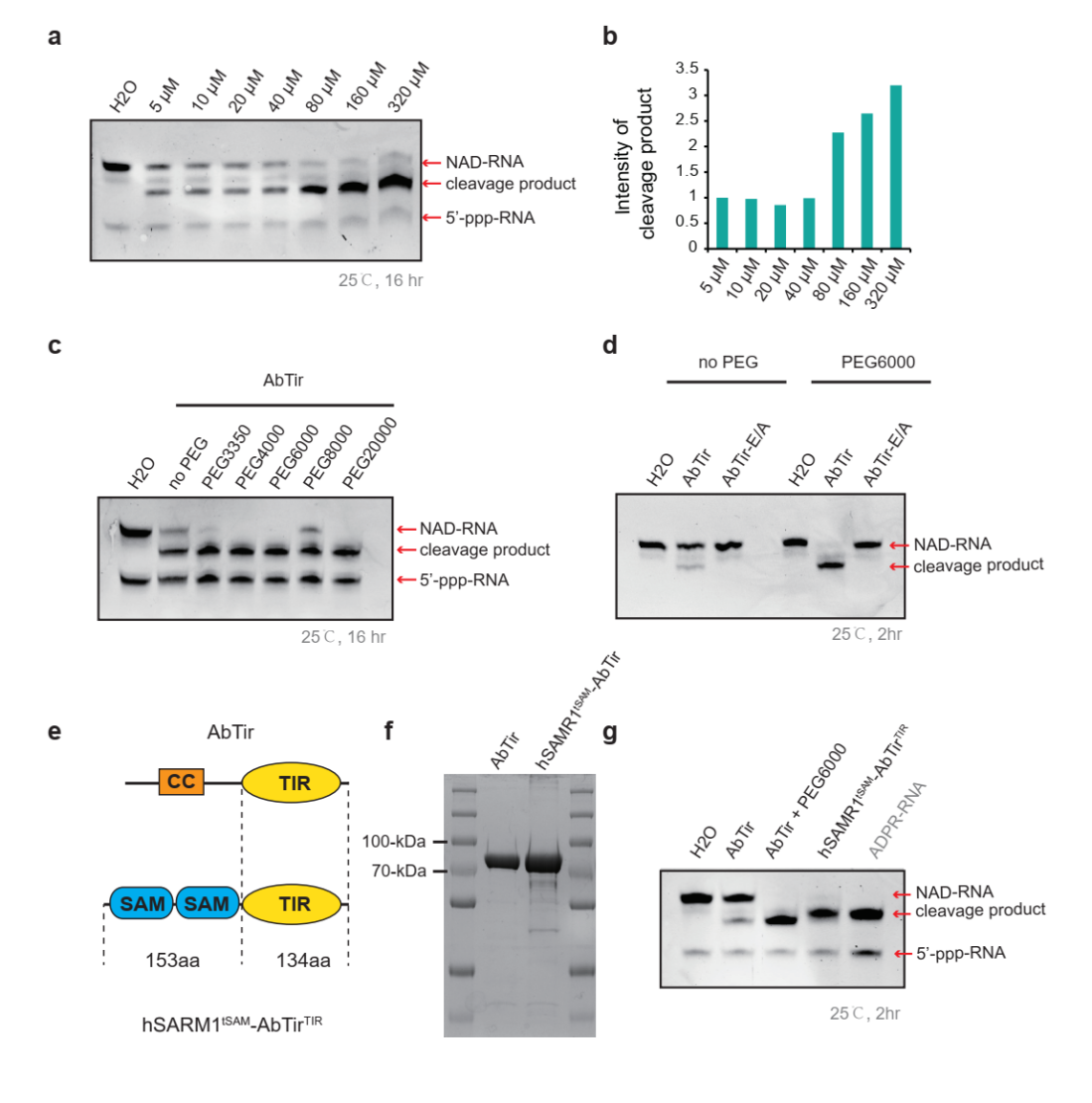


**Supplementary Fig. 5:** Oligomerization enhances the deNAMing activity of AbTir. **a** Analysis of the AbTir deNAMing activity at different enzyme concentrations. After the reactions, RNAs were resolved in an APB gel to separate the deNAMing product from the input NAD-RNA. **b** Quantification of the deNAMing product of AbTir in (a). Relative levels of the deNAMing products are shown (with the level at 5μM AbTir arbitrarily set to 1.0). **c** Effects of macromolecular crowding agents on the AbTir deNAMing activity. **d** PEG6000 significantly increased the deNAMing activity of AbTir, resulting in more NAD-RNA being cleaved under the same reaction conditions (at 25 ℃ for 2 hours). **e** Diagrams of AbTir and the hSARM1^tSAM^-AbTir^TIR^ fusion protein. The tandem SAM domains were cloned from the human SARM1 gene. **f** An SDS-PAGE gel showing the purity of the expressed AbTir and hSARM1^tSAM^-AbTir^TIR^ proteins. The proteins were resolved in a 15% SDS-PAGE gel and stained with Coomassie Brilliant Blue. **g** An APB gel showing the decapping efficiency of hSARM1^tSAM^-AbTir^TIR^ as compared to that of AbTir itself and AbTir with PEG6000 added. Source data are provided as a Source Data file.


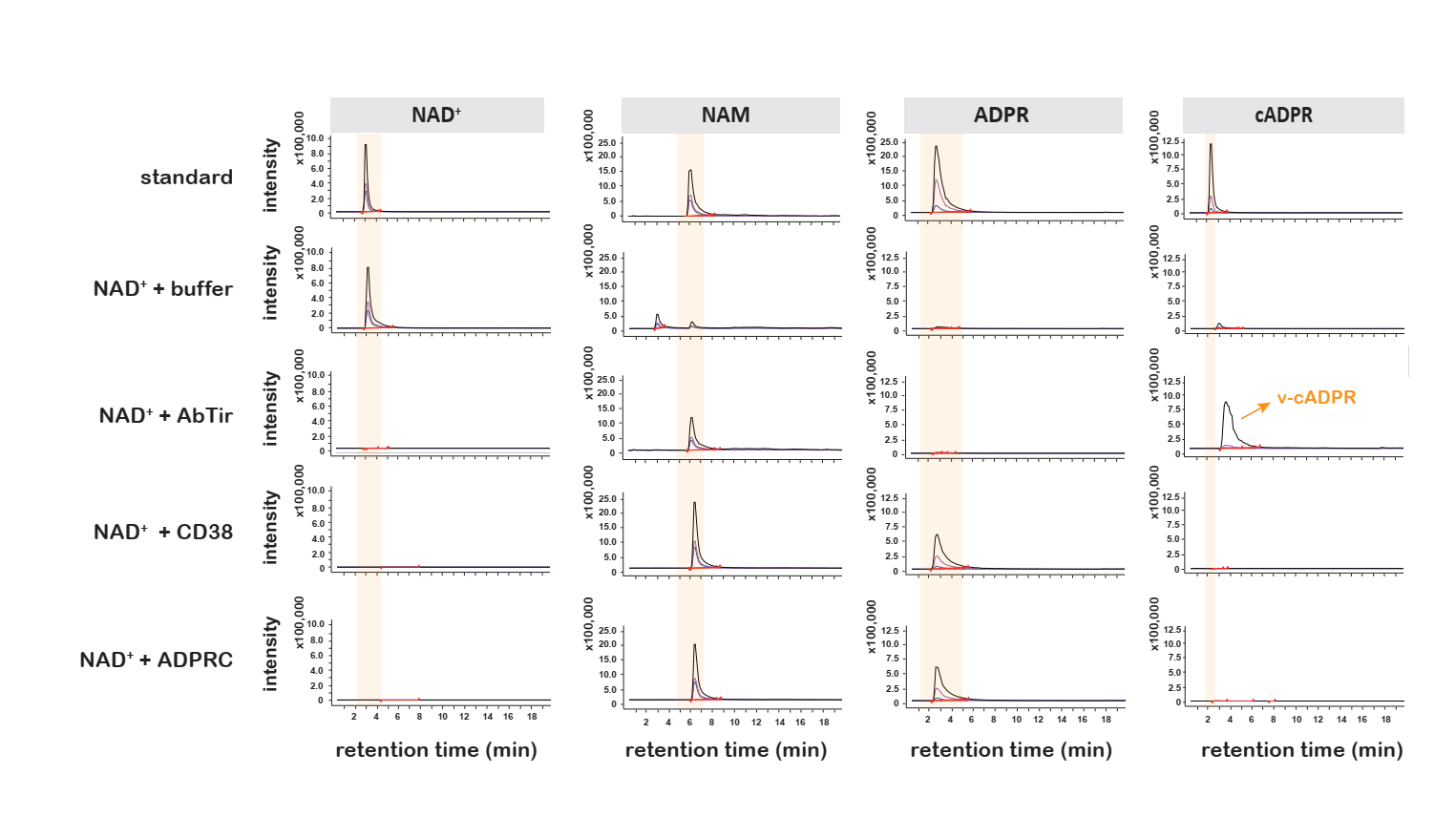


**Supplementary Fig. 6:** LC-MS analysis of products of NADase assays in which AbTir, CD38, and ADPRC were each incubated with NAD^+^. Commercial compounds, including NAD^+^, NAM, ADPR, and cADPR, were used as standards to compare to the reaction products. The product of AbTir is neither ADPR nor cADPR and is likely v-cADPR as reported by Manik et al. (2022) ^[1](#_ENREF_1)^. The three product ions of NAD^+^ ([M+H]^+^ 664.00>136.00, 664.00 >427.90, 664.00>523.95), NAM ([M+H]^+^ 123.00>80.00, 123.00>78.00, 123.00>53.00), cADPR ([M+H]^+^ 541.80>136.15, 541.80>427.90, 541.80>347.90), and ADPR ([M+H]^+^ 559.80>136.05, 559.80>347.95, 559.80>427.95) are indicated by black, pink and blue lines, respectively.


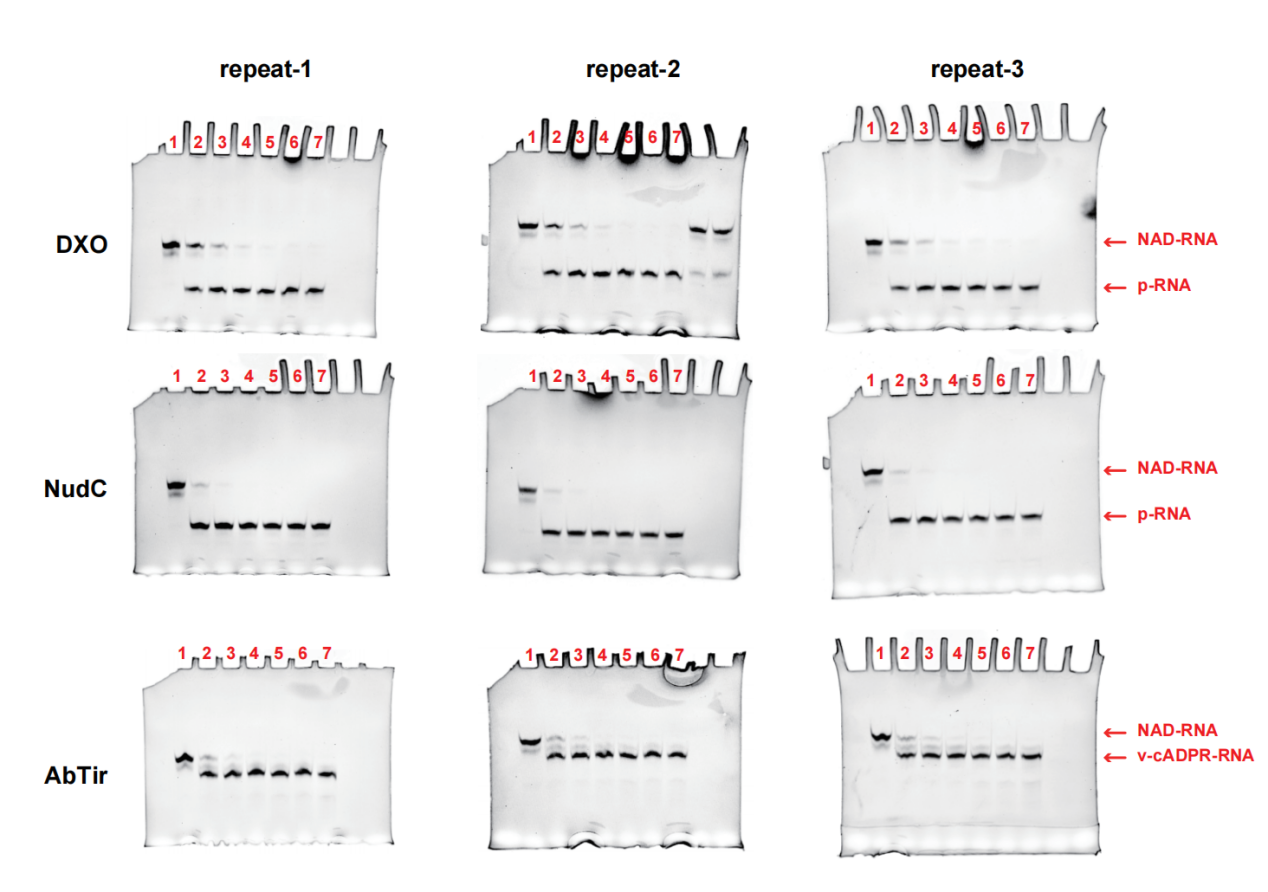


**Supplementary Fig. 7:** Time courses of NAD-RNA decapping assays for NudC, DXO, and AbTir. An *in vitro* transcribed NAD-RNA was incubated with DXO (top), NudC (middle), or AbTir (bottom), and the cleavage products at the different time points were resolved in denaturing APB gels. The time points, which always appear in the same order, are, from left to right: 0 min (1), 10 min (2), 30 min (3), 60 min (4), 90 min (5), 120 min (6), and 150min (7). Three independent experiments (repeats) were performed for each decapping enzyme for the reaction kinetics analysis in **Fig. 4d**.


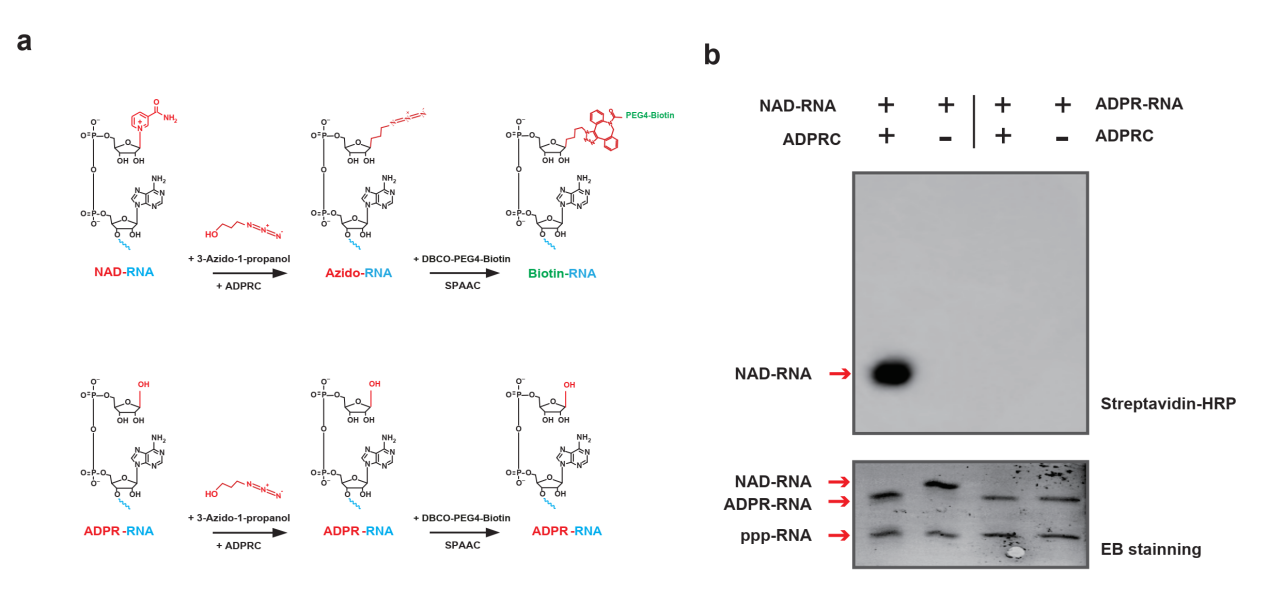


**Supplementary Fig. 8:** ADPR-RNA cannot be biotinylated using the NAD-RNA biotinylation scheme. **a** Pipeline to convert NAD-RNA, but not ADPR-RNA, to biotinylated RNA. **b** An *in vitro* transcribed NAD-RNA or ADPR-RNA was used to perform the pipeline in (a), in which the RNA was incubated with 3-azido-1-propanol in the presence (+) or absence (-) of ADPRC. The reaction products were resolved in a 2% agarose gel and transferred to a nylon N^+^ membrane. Biotin-labeled products were probed with streptavidin–horseradish peroxidase, and signals were detected with a chemiluminescent nucleic acid detection kit. The reaction products were also resolved in a denaturing APB gel and stained with ethidium bromide (EB) to visualize the RNA products.


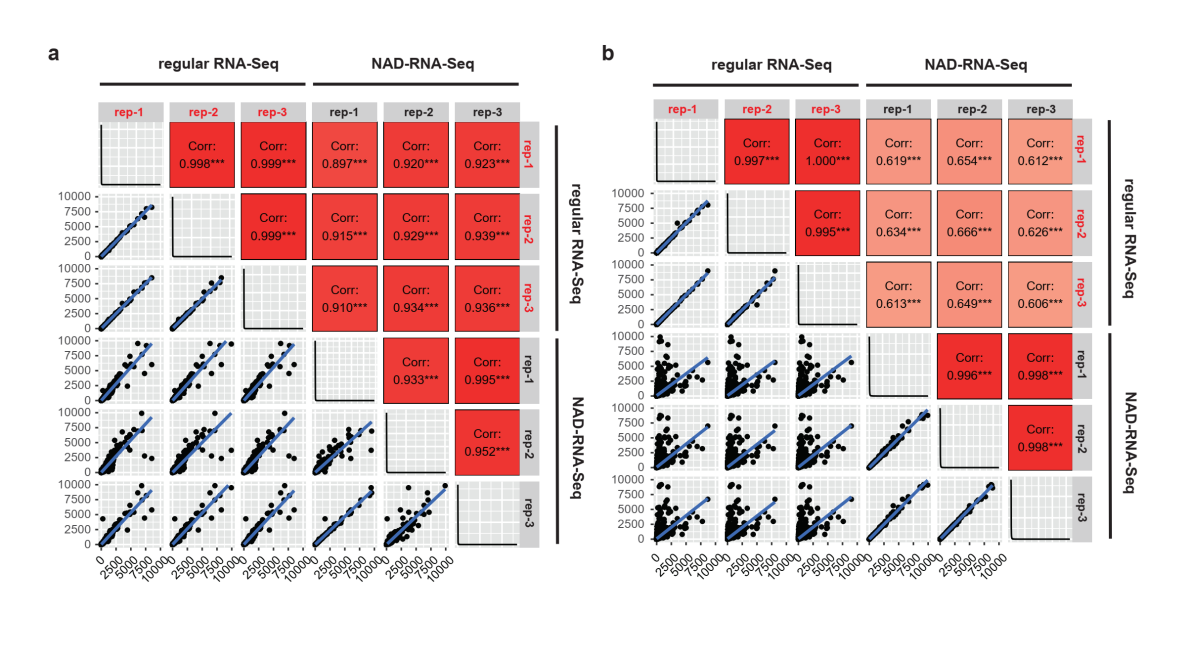


**Supplementary Fig. 9:** Correlation scatter diagrams among biological replicates of SPAAC-NAD-Seq (NAD-RNA-Seq) and regular RNA-Seq prepared with *E. coli* rRNA-depleted RNA after AbTir **(a)** or AbTir-E/A **(b)** treatment. The lower triangular matrices show the scatter plots for RPM (Reads Per Million) comparisons among the biological replicates. The upper triangular matrices indicatesthe correlation coefficients and significance levels among replicates.


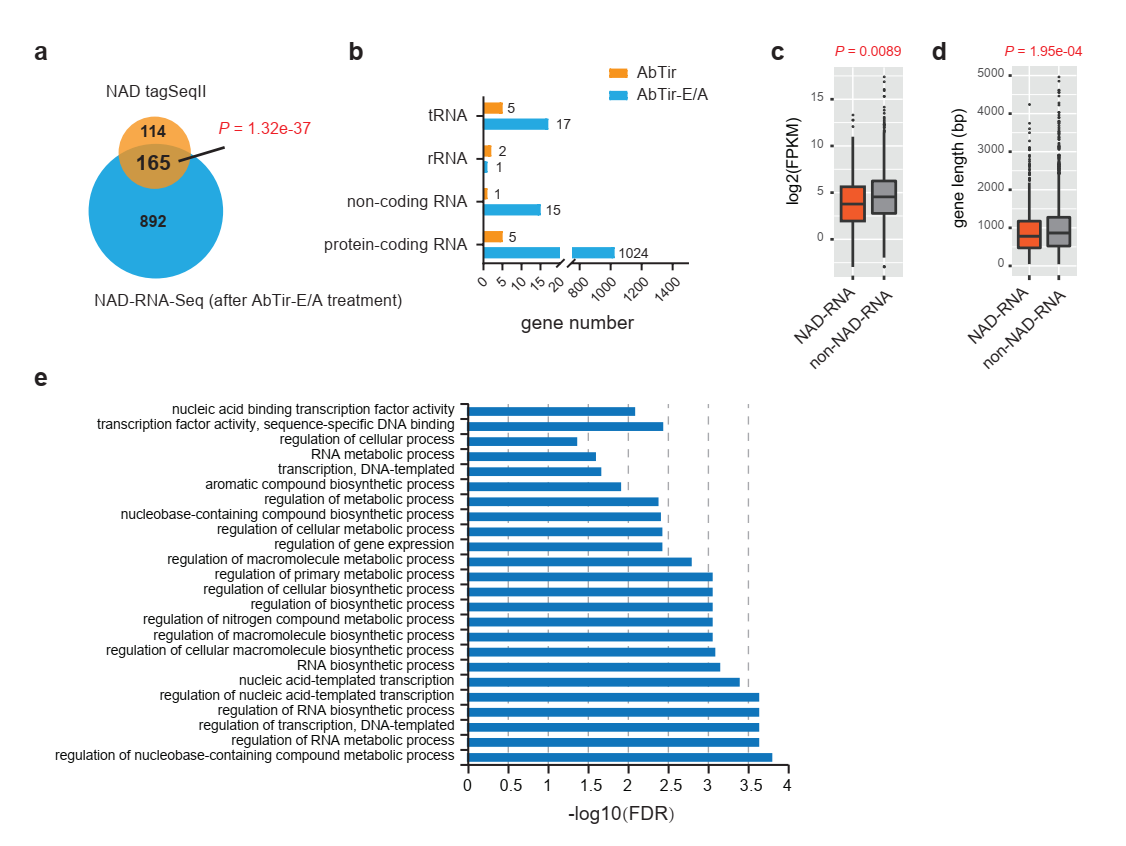


**Supplementary Fig. 10:** Characterization of NAD-RNAs in *E. coli* identified in this study. **a** Venn diagram showing the overlap between NAD-RNA-producing genes identified by Zhang et al with NAD tagSeqII (2021) ^[2](#_ENREF_2)^ and our current study by SPAAC-NAD-Seq. The *P*-value showing the significance of the overlap was calculated by the hypergeometric test. **b** Annotation of NAD-RNA-producing genes identified in BL21 *E. coli* cells after AbTir (yellow) or AbTir-E/A (blue) treatment. **c** Box plot comparing expression levels between genes that produce NAD-RNAs (labeled as “NAD-RNA”) and those that do not (labeled as “non-NAD-RNA”). FPKM, Fragments Per Kilobase Million. **d** Box plot comparing gene lengths between genes that produce NAD-RNAs (labeled as “NAD-RNA”) and those that do not (labeled as “non-NAD-RNA”). Statistical significance levels in **(c)** and **(d)** were calculated by Student’s *t*-test and shown above each plot with red text. **e** Bar plot of Gene Ontology (GO) enrichments of the NAD-RNA-producing genes.


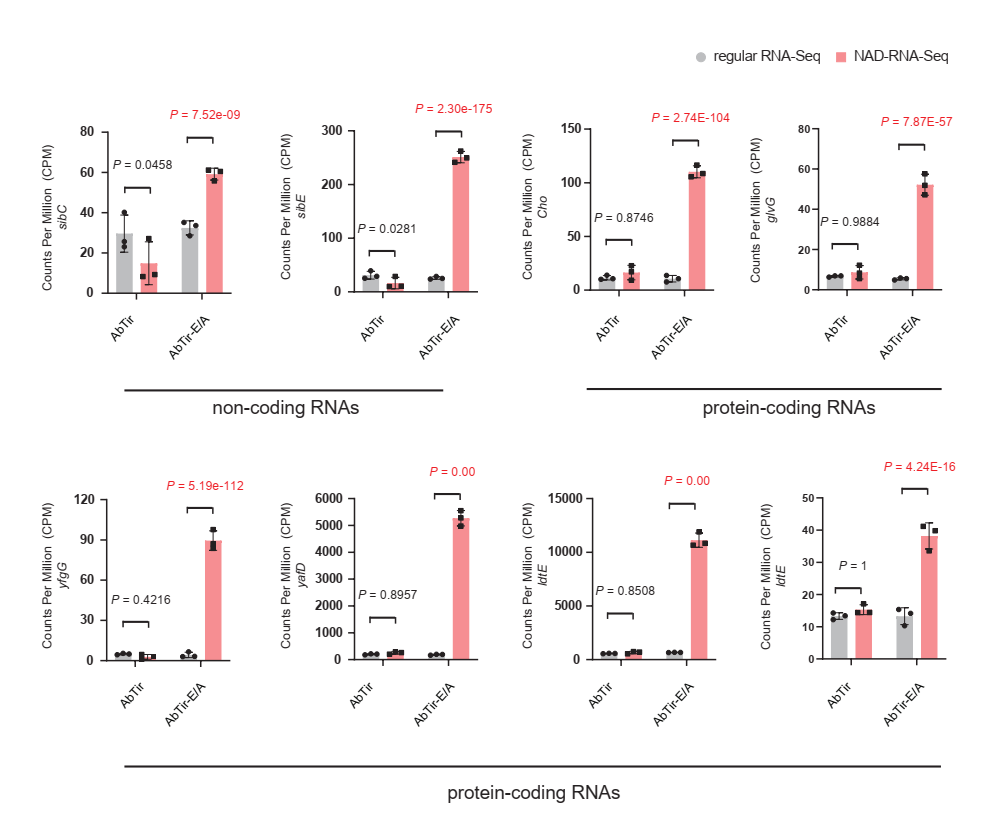


**Supplementary Fig. 11:** NAD-RNA levels from two non-coding RNA genes and six protein-coding genes. These genes were identified as NAD-RNA-producing genes in *E. coli*. After AbTir pretreatment, the NAD-RNA levels were significantly decreased. The *P*-values were calculated by edgeR after FDR multiple correction.


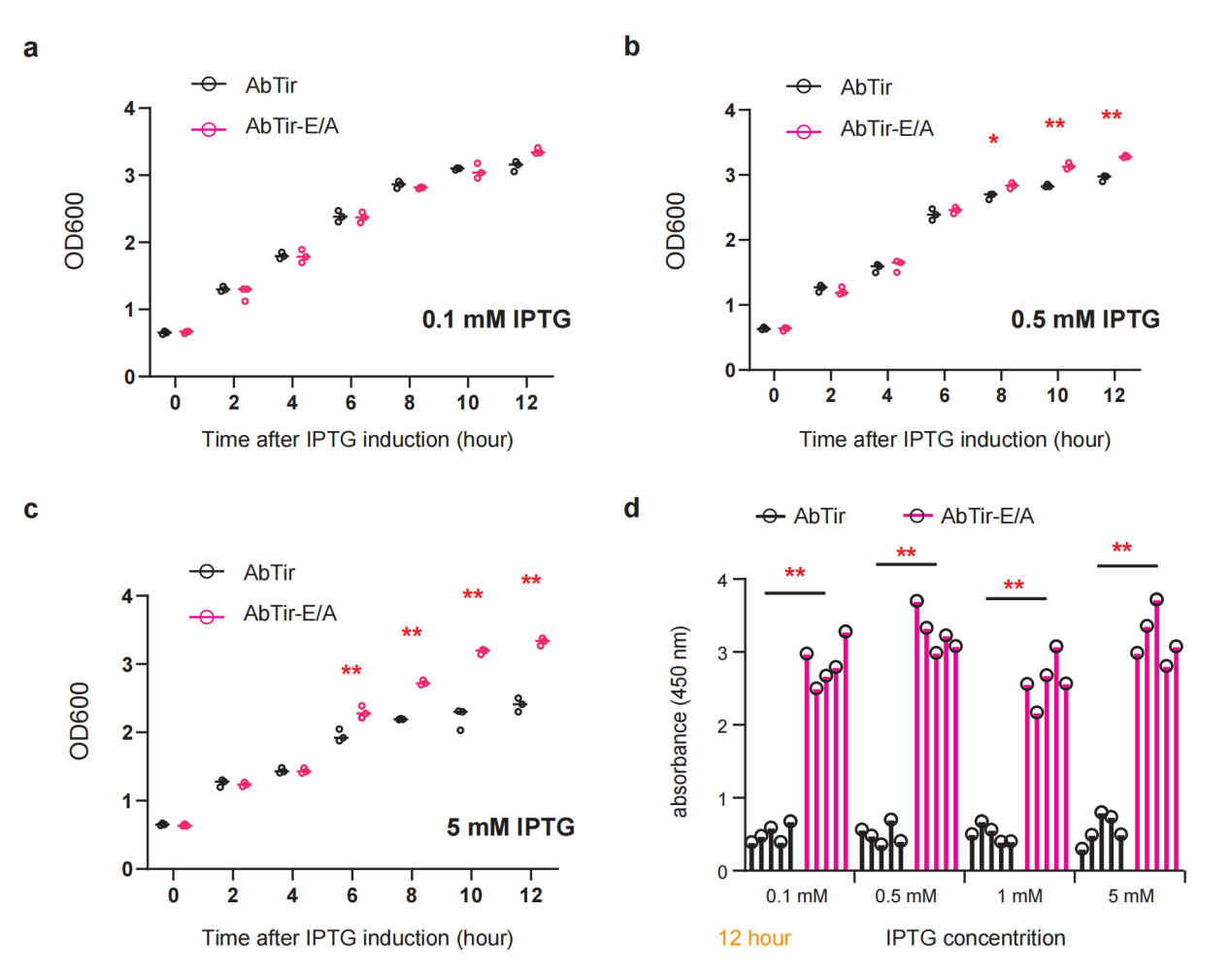


**Supplementary Fig. 12: a-c** OD600 values that reflect the cell growth status at the indicated time points after induction of AbTir or AbTir-E/A expression with 0.1 mM **(a)**, 1 mM **(b)**, or 5 mM **(c)** IPTG. Three biological replicates were measured at each time point (n = 3). [**] *P* ≤ 0.01; [*] *P* ≤ 0.05. *P*-values were calculated by the Student’s t-test (two-sided). **d** Levels of endogenous NAD^+^ in *E. coli* cells expressing AbTir (black) or AbTir-E/A (pink) at 12 hours after induction of AbTir or AbTir-E/A expression with different concentrations of IPTG. Each bar represents a biological replicate and a total of 5 replicates were measured for each IPTG concentration. [**] *P* ≤ 0.01. *P*-values were calculated by the non-parametric Mann-Whitney U-test (two-sided). Exact *P*-values and source data are provided as a Source Data file.


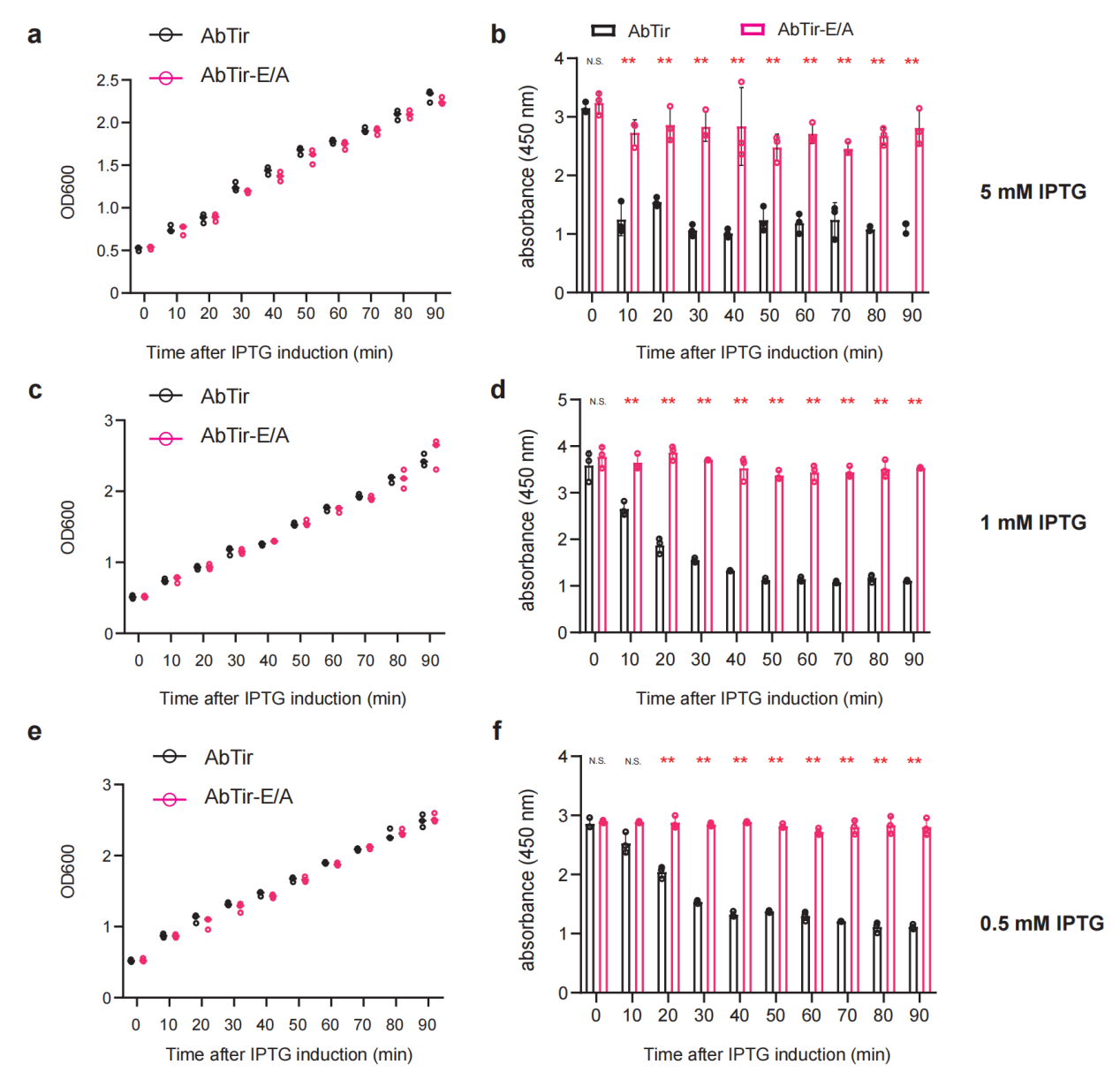


**Supplementary Fig. 13: a, c, e** OD600 values at the indicated time points after addition of 5 mM **(a)**, 1 mM **(b)**, or 0.5 mM **(c)** IPTG for evaluating the cell growth status. Three biological replicates were measured at each time point. **b, d, f** Measurement of NAD^+^ levels in *E. coli* cells expressing AbTir (black) or AbTir-E/A (pink) for the indicated time points after induction by different concentrations of IPTG. Three biological replicates were measured at each time point (n = 3). [**] *P* ≤ 0.01; N.S., not significant (calculated by the Student’s t-test, two-sided). Exact *P*-values and source data are provided as a Source Data file.


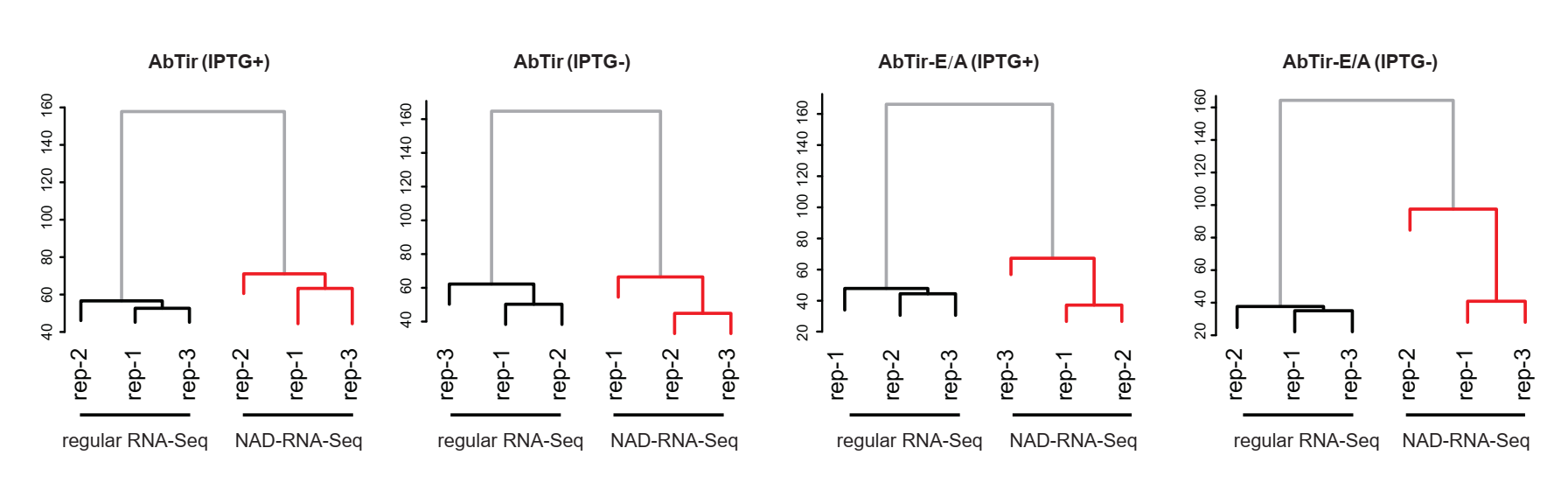


**Supplementary Fig. 14:** Clustering analyses of NAD-RNA-Seq and regular RNA-Seq performed with rRNA-depleted RNA from *E. coli* cells expressing AbTir (AbTir IPTG+) or not (AbTir IPTG-, AbTir –E/A IPTG+, and AbTir-E/A IPTG-). Three biological replicates were prepared for each group.


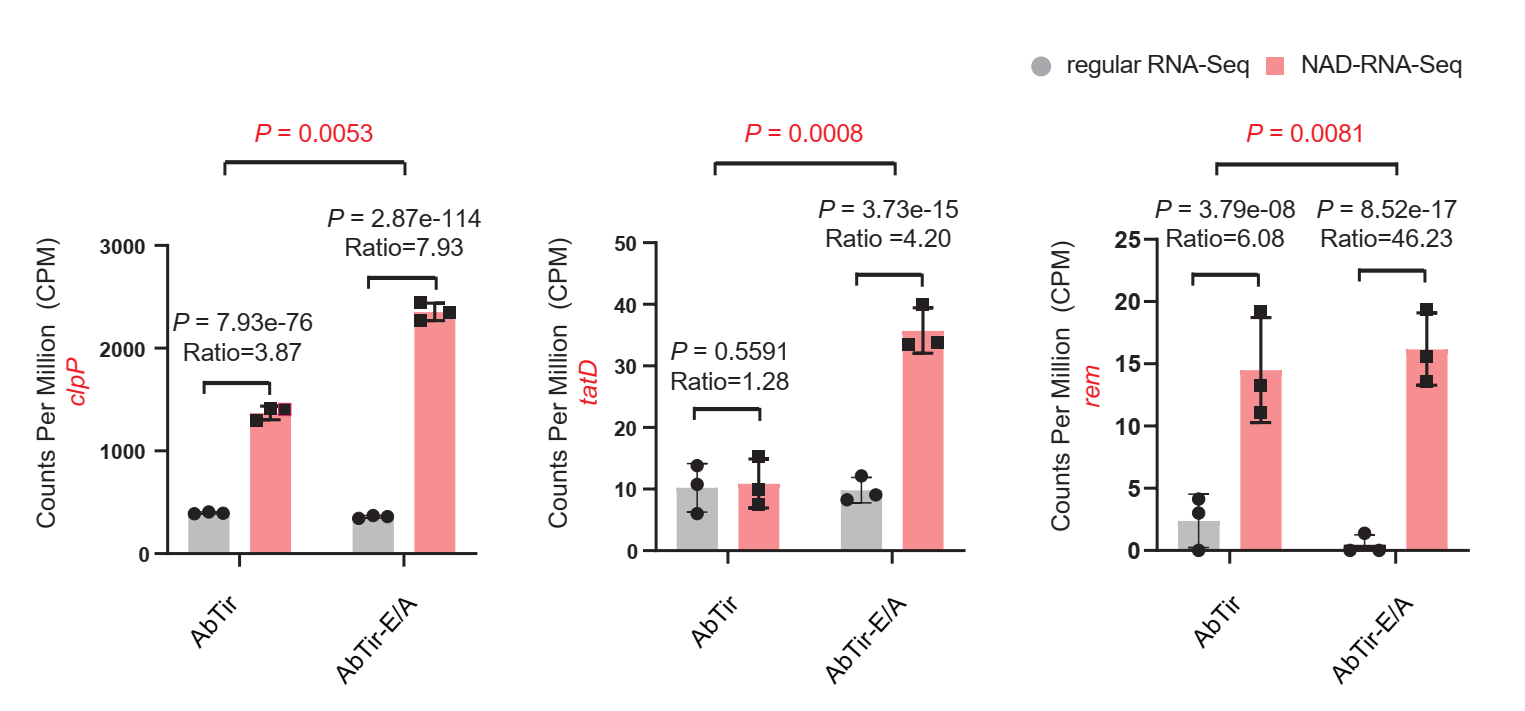


**Supplementary Fig. 15:** Levels of NAD-RNAs from three NAD-RNA-producing genes (*i.e.*, *clpP*, *tatD*, and *rem*) in *E. coli* cells expressing AbTir or AbTir-E/A as determined by SPAAC-NAD-Seq and regular RNA-Seq. The ratio of CPM (NAD-RNA-Seq) *vs.* CPM (regular RNA-Seq) was used to reflect the NAD-RNA level. Genes that pass the filter of “NAD-RNA-Seq/regular RNA-Seq ≥ 2 and FDR ≤ 0.05” were identified as NAD-RNA-producing genes. The *P*-values for NAD-RNA identification (labeled with black font) were calculated by edgeR after FDR multiple correction. The *P*-values for detecting differential NAD-RNA enrichment (labeled with red font) were calculated by Student’s *t*-test.


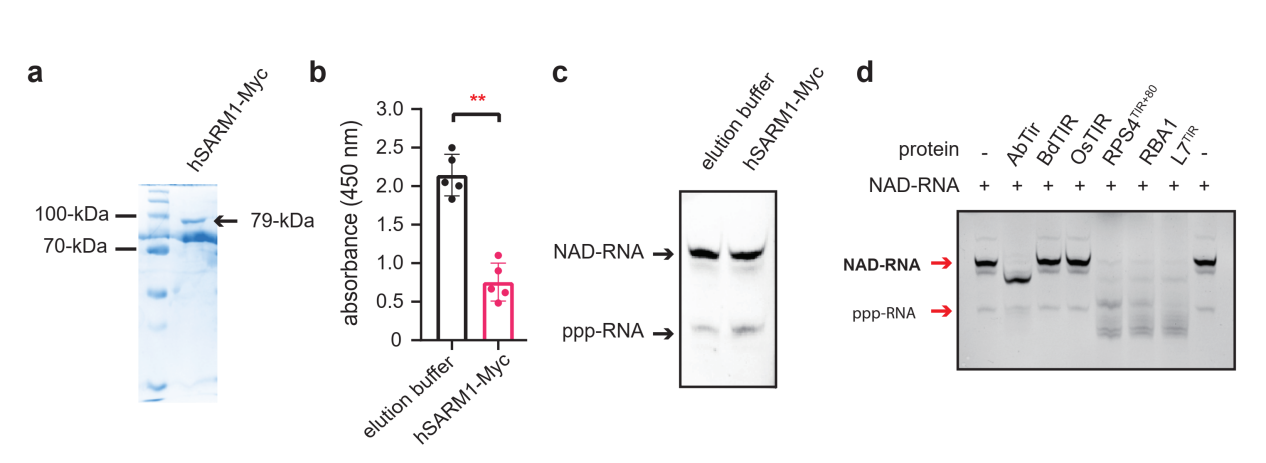


**Supplementary Fig. 16:** Evaluation of the NADase and deNAMing activities of TIR domain-containing proteins in other organisms. **a** An SDS-PAGE gel showing the purity of the commercial hSARM1-myc protein. The protein was resolved in a 15% SDS-PAGE gel and stained with Coomassie Brilliant Blue. **b** NADase activities of hSARM1-myc. **c** An APB gel showing the RNA species after hSARM1 was incubated with an *in vitro* transcribed NAD-RNA. **d** An APB gel showing the RNA species after various TIR domain proteins from plants were incubated with an *in vitro* transcribed NAD-RNA. The reaction with AbTir was included as a positive control. The *In vitro* transcribed NAD-RNA was also loaded as a maker on the APB gel. The y-axes in **(b)** represent relative NAD^+^ levels, which were measured with the NAD/NADH Quantitation Kit by monitoring the absorption values at 450 nm. Error bars represent mean ± SD, which was calculated from five independent experiments (n = 5); [**] *P* ≤ 0.01 (calculated by the non-parametric Mann-Whitney U-test, two-sided). N.S., not significant. Exact *P-*values and source data are provided as a Source Data file.


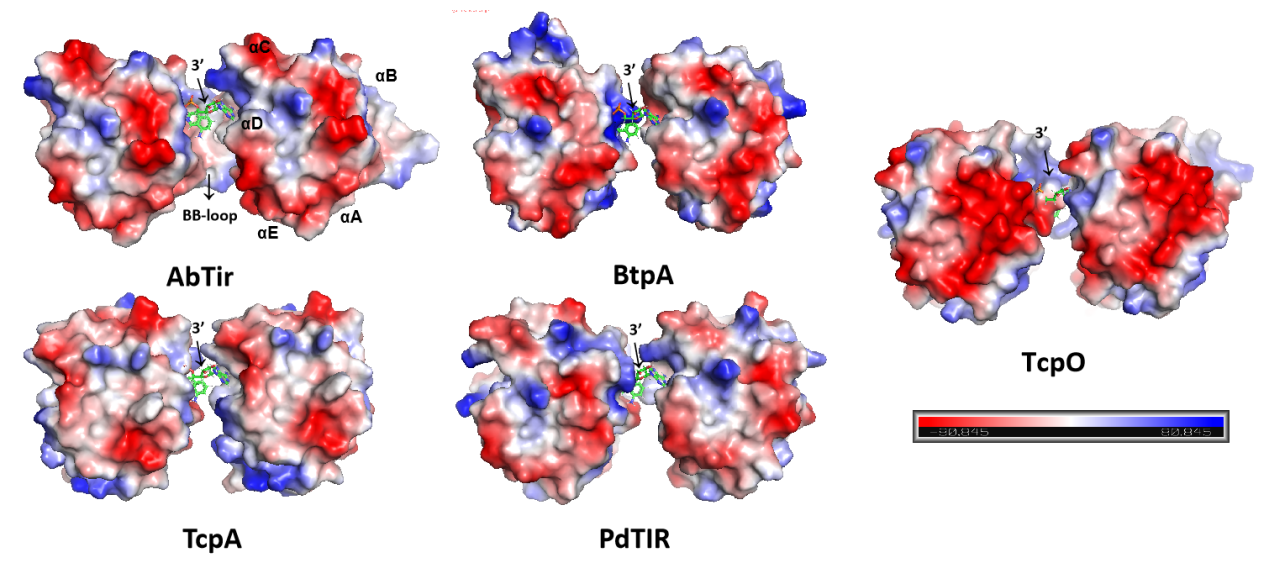


**Supplementary Fig. 17: Surface charges of various TIR-domain proteins.** The cryo-EM structure of the asymmetric dimer from the TIR domain of AbTir (7UXU) is shown in surface representation, with electrostatic potential mapped to the surface. The 3’ position of 8-amino-isoquinoline adenine dinucleotide (3AD) bound in the catalytic center is labeled. Structures of the TIR domains of TcpA, BtpA and TcpO were predicted by AlphaFold2 ^3^. TcpA, BtpA and TcpO and PdTIR (3H16) were superimposed onto the AbTir asymmetric dimer. Blue and red colors indicate the positive and negative charges on the protein surface.

**Supplementary Table 1:** Primers used for plasmid construction and PCR-mediated site-directed mutagenesis.

| **Primer ID** | **Primer Sequences (5’->3’)** |
| --- | --- |
| AbTIR-pMAL-BamH1-F | GTTCTGTTCCAGGGGCCCCATATGGGATCCATGAGCCTTGAGCAGAAGAAG |
| AbTIR-pMAL-Xho1-R | ACGGCCAGTGCCAAGCTTTCACTCGAGTTAATGATGATGGTGATGATGCCTGTTCAGAATCACATCAGCC |
| BtTIR-pMAL-BamH1-F | GTTCTGTTCCAGGGGCCCCATATGGGATCCATGAATTCATCTTACTATCAA |
| BtTIR-pMAL-Xho1-R | ACGGCCAGTGCCAAGCTTTCACTCGAGTTAATGATGATGGTGATGATGTAAATTCTTCAGGTTTTCGAC |
| TcpC-pMAL-BamH1-F | GTTCTGTTCCAGGGGCCCCATATGGGATCCTTTTTCATATCCCATGCAAAAGAG |
| TcpC-pMAL-Xho1-R | ACGGCCAGTGCCAAGCTTTCACTCGAGTTAATGATGATGGTGATGATGTCTTCTCCTGTATGCTATTTC |
| BtpA-pMAL-BamH1-F | GTTCTGTTCCAGGGGCCCCATATGGGATCCATGAGTTCGTACTCTTCTAAT |
| BtpA-pMAL-Xho1-R | ACGGCCAGTGCCAAGCTTTCACTCGAGTTAATGATGATGGTGATGATGGATAAGGGAATGCAGTTCTTT |
| TcpF-pMAL-BamH1-F | GTTCTGTTCCAGGGGCCCCATATGGGATCCGTGAGCAACGGGAAAAAGAT |
| TcpF-pMAL-Xho1-R | ACGGCCAGTGCCAAGCTTTCACTCGAGTTAATGATGATGGTGATGATGCTCTACCTTCTCTAAATAAGA |
| PdTIR-pMAL-BamH1-F | GTTCTGTTCCAGGGGCCCCATATGGGATCCATGAGCGCGAATGATCGAGCA |
| PdTIR-pMAL-Xho1-R | ACGGCCAGTGCCAAGCTTTCACTCGAGTTAATGATGATGGTGATGATGATCTCTGATAATCGCCATAAG |
| TirS-pMAL-BamH1-F | GTTCTGTTCCAGGGGCCCCATATGGGATCCATGAGCGCGAATGATCGAGCAATA |
| TirS-pMAL-Xho1-R | ACGGCCAGTGCCAAGCTTTCACTCGAGTTAATGATGATGGTGATGATGTCAATCTCTGATAATCGCCATAAG |
| TcpA-pMAL-BamH1-F | GTTCTGTTCCAGGGGCCCCATATGGGATCCATGGCGAAGAGTGTTTCCATT |
| TcpA-pMAL-Xho1-R | ACGGCCAGTGCCAAGCTTTCACTCGAGTTAATGATGATGGTGATGATGGACAGACTTCTTTCTGAGAAC |
| TcpO-pMAL-BamH1-F | GTTCTGTTCCAGGGGCCCCATATGGGATCCATGGAGGATTTAGAAATTTTT |
| TcpO-pMAL-Xho1-R | ACGGCCAGTGCCAAGCTTTCACTCGAGTTAATGATGATGGTGATGATGAGATTTTTCCTCATTTATACG |
| RPS4-TIRpMAL-BamH1-F | GTTCTGTTCCAGGGGCCCCATATGGGATCCATGGAGACATCATCTATTTCC |
| RPS4-TIR80-pMAL-Xho1-R | ACGGCCAGTGCCAAGCTTTCACTCGAGTTAATGATGATGGTGATGATGTTGAGGCAAGCGATCCAACTC |
| RBA1-GST-F | CTGTACTTCCAATCCAATATGACGAGCGTGTCTCCTCGG |
| RBA1-GST-R | GTCGACGGAGCTCGAATTTCAAATCCTTACAGTCCTGTC |
| BdTIR-pMAL-BamH1-F | GTTCTGTTCCAGGGGCCCCATATGGGATCCATGGCGTCGTCGGGGCTTTCT |
| BdTIR-pMAL-Xho1-R | ACGGCCAGTGCCAAGCTTTCACTCGAGTTAATGATGATGGTGATGATGGAGCCTTGAAAGGATCATCGT |
| OsTIR-pMAL-BamH1-F | GTTCTGTTCCAGGGGCCCCATATGGGATCCATGAGCAGCACCGGTCTGAGCCGT |
| OsTIR-pMAL-Xho1-R | ACGGCCAGTGCCAAGCTTTCACTCGAGTTAATGATGATGGTGATGATGCAGACGGCTCATAATCATGTG |
| AbTIR-E208A-F | ACTGGACCAACTACGCGCTGGATGGT |
| AbTIR-E208A-R | GCGTAGTTGGTCCAGTCTTTCTTGAT |
| PdTIR-E239A-F | AGTGGCCTCAGAAAGCGCTGGACGGG |
| PdTIR-E239A-R | GCTTTCTGAGGCCACTCCTTCTTGAA |
| BtpA-E217A-F | AATGGCCCGCAAGAGCATTAGATGGA |
| BtpA-E217A-R | GCTCTTGCGGGCCATTGCTTGCTAAA |
| TcpA-E267A-F | AATGGCCCCAAAAAGCACTGGATGGT |
| TcpA-E267A-R | GCTTTTTGGGGCCATTCTTTTTCAAA |
| TcpO-E279A-F | AATGGACTAATTATGCATATGATAAC |
| TcpO-E279A-R | GCATAATTAGTCCATTTACTTTTAAA |
| AbTIR-W204A-F | TCATCAAGAAAGACGCGACCAACTAC |
| AbTIR-W204A-R | GCGTCTTTCTTGATGAAGTCGGTGCT |

**Supplementary Data 1:** NAD-RNA-producing genes identified by SPAAC-NAD-Seq in *E. coli* RNA after AbTir pre-treatment *in vitro*.

**Supplementary Data 2:** NAD-RNA-producing genes identified by SPAAC-NAD-Seq in *E. coli* RNA after AbTir-E/A pre-treatment *in vitro*.

**Supplementary Data 3:** NAD-RNA-producing genes identified by SPAAC-NAD-Seq in *E. coli* cells that harbor the AbTir plasmid and were treated with IPTG to induce AbTir expression.

**Supplementary Data 4:** NAD-RNA-producing genes identified by SPAAC-NAD-Seq in *E. coli* cells that harbor the AbTir plasmid but were not treated with IPTG.

**Supplementary Data 5:** NAD-RNA-producing genes identified by SPAAC-NAD-Seq in *E. coli* cells that harbor the AbTir-E/A plasmid were treated with IPTG to induce AbTir-E/A expression.

**Supplementary Data 6:** NAD-RNA-producing genes identified by SPAAC-NAD-Seq in *E. coli* cells that harbor the AbTir-E/A plasmid but were not treated with IPTG.

**References:**

1. Manik, M.K. *et al.* Cyclic ADP ribose isomers: Production, chemical structures, and immune signaling. *Science* **377**, eadc8969 (2022).

2. Zhang, H. *et al.* Use of NAD tagSeq II to identify growth phase-dependent alterations in *E. coli* RNA NAD^+^ capping. *Proceedings of the National Academy of Sciences* **118**, e2026183118 (2021).

3. Jumper, J. *et al*. Highly accurate protein structure prediction with AlphaFold. *Nature* **596**,583- 589 (2021).
