# Supplementary material for: Toll/interleukin-1 receptor (TIR) domain-containing proteins have NAD-RNA decapping activity
Source: Nat Commun. 2024 Mar 13;15:2261. doi: 10.1038/s41467-024-46499-y (PMC10937652; doi:10.1038/s41467-024-46499-y)
Supplement: Supplementary file 3 — Description of Additional Supplementary Files [file 41467_2024_46499_MOESM3_ESM.pdf]

Description of Additional Supplementary Files

File Name: Supplementary Data 1
Description: NAD-RNA-producing genes identified by SPAAC-NAD-Seq in *E. coli* RNA after AbTir pre-treatment *in vitro.*

File Name: Supplementary Data 2
Description: NAD-RNA-producing genes identified by SPAAC-NAD-Seq in *E. coli* RNA after AbTir-E/A pre-treatment *in vitro.*

File Name: Supplementary Data 3
Description: NAD-RNA-producing genes identified by SPAAC-NAD-Seq in *E. coli* cells that harbor the AbTir plasmid and were treated with IPTG to induce AbTir expression.

File Name: Supplementary Data 4
Description: NAD-RNA-producing genes identified by SPAAC-NAD-Seq in *E. coli* cells that harbor the AbTir plasmid but were not treated with IPTG.

File Name: Supplementary Data 5
Description: NAD-RNA-producing genes identified by SPAAC-NAD-Seq in *E. coli* cells that harbor the AbTir-E/A plasmid were treated with IPTG to induce AbTir-E/A expression.

File Name: Supplementary Data 6
Description: NAD-RNA-producing genes identified by SPAAC-NAD-Seq in *E. coli* cells that harbor the AbTir-E/A plasmid but were not treated with IPTG.
