# Supplementary material for: Toll/interleukin-1 receptor (TIR) domain-containing proteins have NAD-RNA decapping activity
Source: Nat Commun. 2024 Mar 13;15:2261. doi: 10.1038/s41467-024-46499-y (PMC10937652; doi:10.1038/s41467-024-46499-y)
Supplement: Supplementary file 10 — Reporting Summary [file 41467_2024_46499_MOESM10_ESM.pdf]

nature portfolio

Corresponding author(s): Xuemei Chen

Last updated by author(s): Feb 21, 2024

Reporting Summary

Nature Portfolio wishes to improve the reproducibility of the work that we publish. This form provides structure for consistency and transparency in reporting. For further information on Nature Portfolio policies, see our Editorial Policies and the Editorial Policy Checklist.

Statistics

For all statistical analyses, confirm that the following items are present in the figure legend, table legend, main text, or Methods section.

n/a	Confirmed	
☐	☒	The exact sample size (*n*) for each experimental group/condition, given as a discrete number and unit of measurement
☐	☒	A statement on whether measurements were taken from distinct samples or whether the same sample was measured repeatedly
☐	☒	The statistical test(s) used AND whether they are one- or two-sided<br>*Only common tests should be described solely by name; describe more complex techniques in the Methods section.*
☒	☐	A description of all covariates tested
☐	☒	A description of any assumptions or corrections, such as tests of normality and adjustment for multiple comparisons
☐	☒	A full description of the statistical parameters including central tendency (e.g. means) or other basic estimates (e.g. regression coefficient) AND variation (e.g. standard deviation) or associated estimates of uncertainty (e.g. confidence intervals)
☐	☒	For null hypothesis testing, the test statistic (e.g. *F*, *t*, *r*) with confidence intervals, effect sizes, degrees of freedom and *P* value noted<br>*Give P values as exact values whenever suitable.*
☒	☐	For Bayesian analysis, information on the choice of priors and Markov chain Monte Carlo settings
☒	☐	For hierarchical and complex designs, identification of the appropriate level for tests and full reporting of outcomes
☒	☐	Estimates of effect sizes (e.g. Cohen's *d*, Pearson's *r*), indicating how they were calculated

Our web collection on statistics for biologists contains articles on many of the points above.

Software and code

Policy information about availability of computer code

Data collection	The LCMS-8060 triple quadrupole mass spectrometer with electro spray ionization (Shimadzu) and scheduled multiple reaction monitoring (MRM) was employed to monitor analyte parent ions to product ion formation. The absorbance was measured at 450nm using SpectraMax iD3@ Multi-mode microplate reader (Sunnyvale, CA, USA).
Data analysis	R software and the corresponding packages were used for NAD-RNA identification, differential NAD-RNA level analysis, statistical analysis, and figure preparation. GraphPad Prism 9 software was used for statistical analysis to examine significant differences between two groups.

For manuscripts utilizing custom algorithms or software that are central to the research but not yet described in published literature, software must be made available to editors and reviewers. We strongly encourage code deposition in a community repository (e.g. GitHub). See the Nature Portfolio guidelines for submitting code & software for further information.

Data

Policy information about availability of data

All manuscripts must include a data availability statement. This statement should provide the following information, where applicable:

- Accession codes, unique identifiers, or web links for publicly available datasets
- A description of any restrictions on data availability
- For clinical datasets or third party data, please ensure that the statement adheres to our policy

The clean data of regular RNA-Seq and NAD-RNA-Seq in this study have been deposited in National Center for Biotechnology Information (NCBI) (BioProject database, accession code PRJNA1061094).

Research involving human participants, their data, or biological material

Policy information about studies with human participants or human data. See also policy information about sex, gender (identity/presentation), and sexual orientation and race, ethnicity and racism.

Reporting on sex and gender	N/A
Reporting on race, ethnicity, or other socially relevant groupings	N/A
Population characteristics	N/A
Recruitment	N/A
Ethics oversight	N/A

Note that full information on the approval of the study protocol must also be provided in the manuscript.

Field-specific reporting

Please select the one below that is the best fit for your research. If you are not sure, read the appropriate sections before making your selection.

☒ Life sciences ☐ Behavioural & social sciences ☐ Ecological, evolutionary & environmental sciences

For a reference copy of the document with all sections, see nature.com/documents/nr-reporting-summary-flat.pdf

Life sciences study design

All studies must disclose on these points even when the disclosure is negative.

Sample size	We used at least 3 independent experiments for the comparative analyses of the NADase assays to obtain the mean and standard deviation. The P-values were calculated by the parametric student's t-test or non-parametric Mann-Whitney U-test (two-sided). Three replicates were used for regular RNA-Seq or SPAAC-NAD-Seq. The sample size in each experiment is enough to perform the comparative test, as is commonly used.
Data exclusions	No data were excluded from the analyses
Replication	As in the RNA-Seq and SPAAC-NAD-Seq analyses, we performed correlation and clustering analyses to check the reproducibility (See Supplementary Figures). We also compared the identified NAD-RNA with the known NAD-RNA producing genes reported in a previous paper, and found that the two datasets overlapped well (Figure 5).
Randomization	The bacterial cells expressing AbTir or AbTir-E/A were separately prepared with three biological replicates for each group. The samples were not randomized during library preparation.
Blinding	The investigators were not blinded to AbTir or AbTir-E/A expressing cells.

Reporting for specific materials, systems and methods

We require information from authors about some types of materials, experimental systems and methods used in many studies. Here, indicate whether each material, system or method listed is relevant to your study. If you are not sure if a list item applies to your research, read the appropriate section before selecting a response.

Materials & experimental systems

n/a	Involved in the study	
☐	☒	Antibodies
☒	☐	Eukaryotic cell lines
☒	☐	Palaeontology and archaeology
☐	☒	Animals and other organisms
☒	☐	Clinical data
☒	☐	Dual use research of concern
☒	☐	Plants

Methods

n/a	Involved in the study	
☒	☐	ChIP-seq
☒	☐	Flow cytometry
☒	☐	MRI-based neuroimaging

Antibodies

Antibodies used	mouse Anti-MBP Monoclonal Antibody (NEB E8032S);
Validation	This antibody is commerical and widely used. In our experiments, the N-termini of AbTir or AbTir-E/A are fused with a known MBP tag. So, it is easy to judge if the antibody worked from the molecular weight of the fusion proteins as well as by comparison to negative controls. The Anti-MBP was diluted 1:5000.

Animals and other research organisms

Policy information about studies involving animals; ARRIVE guidelines recommended for reporting animal research, and Sex and Gender in Research

Laboratory animals	N/A
Wild animals	N/A
Reporting on sex	N/A
Field-collected samples	N/A
Ethics oversight	N/A

Note that full information on the approval of the study protocol must also be provided in the manuscript.

Plants

Seed stocks	N/A
Novel plant genotypes	N/A
Authentication	N/A
